# Damage Detection and Localization under Variable Environmental Conditions Using Compressed and Reconstructed Bayesian Virtual Sensor Data

**DOI:** 10.3390/s22010306

**Published:** 2021-12-31

**Authors:** Jyrki Kullaa

**Affiliations:** Department of Automotive and Mechanical Engineering, Metropolia University of Applied Sciences, Leiritie 1, 01600 Vantaa, Finland; jyrki.kullaa@metropolia.fi

**Keywords:** data compression, data reconstruction, virtual sensing, damage detection, damage localization, optimal sensor placement, environmental effects, whitening, spatiotemporal correlation, time domain

## Abstract

Structural health monitoring (SHM) with a dense sensor network and repeated vibration measurements produces lots of data that have to be stored. If the sensor network is redundant, data compression is possible by storing the signals of selected Bayesian virtual sensors only, from which the omitted signals can be reconstructed with higher accuracy than the actual measurement. The selection of the virtual sensors for storage is done individually for each measurement based on the reconstruction accuracy. Data compression and reconstruction for SHM is the main novelty of this paper. The stored and reconstructed signals are used for damage detection and localization in the time domain using spatial or spatiotemporal correlation. Whitening transformation is applied to the training data to take the environmental or operational influences into account. The first principal component of the residuals is used to localize damage and also to design the extreme value statistics control chart for damage detection. The proposed method was studied with a numerical model of a frame structure with a dense accelerometer or strain sensor network. Only five acceleration or three strain signals out of the total 59 signals were stored. The stored and reconstructed data outperformed the raw measurement data in damage detection and localization.

## 1. Introduction

Structural health monitoring (SHM) is based on frequent vibration measurements using a sensor network with a large number of sensors. As a result, the amount of data is tremendous. An increasing number of sensors is anticipated in future applications—for example, sensing skins [1]. The advantage of a dense sensor network is more reliable damage identification. A disadvantage is higher hardware and data management costs. Historical data must be stored for several years. These training data are used to capture the dynamic characteristics of the undamaged structure under variable environmental or operational conditions. The future measurements will be compared with the training data in order to detect and localize damage.

Data storage may be costly or the amount of data may exceed the storage capacity. Therefore, data reduction is necessary. One method for data reduction is to extract and store only selected features from the time records. Features are dynamic characteristics of the structure, which are expected to be sensitive to damage. Such features are, for example, natural frequencies and mode shapes, which can be extracted from the measurement data using system identification techniques [2]. Significant data compression is possible, because each measurement yields a single data point. However, the original time histories will be lost and cannot be recovered. Therefore, it may be necessary to save everything, resulting in terabytes of data every day [3].

The most common dimensionality reduction method is principal component analysis (PCA) [4], which is a linear method that maximizes the variance in the data by projecting the multidimensional data onto directions, principal components (PC) that account for the largest variability. If only a few PCs are retained, some loss of data results in the reconstruction. PCA has been applied, e.g., to image compression [5]. A disadvantage of PCA is that the reconstruction error is not available. Therefore, it may be difficult to select the number of PCs for storage. If the chosen number of PCs exceeds the optimum, the reconstruction error increases, because the PCs start to model the noise [6].

It is also possible to select a limited number of sensor signals that are permanently stored so that the omitted signals can be accurately reconstructed [6]. The selection is related to optimal sensor placement (OSP), which has been investigated in many applications [7]. Although the objective in those applications is to place a limited number of physical sensors in optimal positions, the same approach can be used to select a subset of signals for storage. The selection in this paper is based on the maximum accuracy of the reconstructed signals.

Some review papers and comparisons of different optimal sensor placement (OSP) algorithms exist [8,9,10,11]. They present the most commonly applied algorithms and criteria. The sensor placement is a discrete optimization problem for which genetic algorithms have been proposed [12,13,14]. Alternatively, a computationally efficient and widely used algorithm is to start with a large set of candidate sensor locations and remove one sensor in each round based on the selected cost function until the stopping criterion is met. This backward sequential sensor placement (BSSP) algorithm has been used in many studies [15,16,17]. Another iterative method is to add one sensor in turn to the sensor network until the stopping criterion is met. The algorithm is called the forward sequential sensor placement (FSSP) algorithm [12,17]. BSSP is used in this study, because experiments have shown its better performance over FSSP.

Data compression results in a decrease in accuracy of the reconstructed signals. Since the signal-to-noise ratio (SNR) is important for damage detection [18], data compression may also decrease the performance of the SHM system. The main novelty of the present paper is to introduce a data compression technique so that the data compression ratio is large and the reconstructed data are so accurate that they can be applied to SHM. With empirical Bayesian virtual sensing, the measurement error can be decreased before data compression so that the stored and reconstructed signals are more accurate than the original measurements. Proof is given that it is more beneficial to store selected virtual sensor signals than the corresponding physical sensor signals [6].

The objective of the present study is to detect and localize damage using the stored and reconstructed virtual sensor data. Damage detection is based on changes in the dynamic characteristics of the structure. Records of structural motion, for example, acceleration or strain, are measured simultaneously at selected degrees of freedom. First, a training data set is acquired from the undamaged structure under different environmental or operational conditions. These data are used to build a statistical data model of the undamaged structure. Next, the structure is monitored with repeated measurements in order to have an early warning of structural failure. The new data are compared to the training data using novelty detection techniques, and a statistically significant change in the dynamic characteristics is an indication of damage. Particular attention is needed to take different environmental or operational conditions into account, because they can have a considerable influence on the very same dynamic characteristics. Several techniques have been proposed to eliminate the environmental or operational influences on the data, even without measuring the underlying quantities; see, e.g., [19,20] and the references therein. Another novelty of this paper is the application of whitening transformation [21] to the training data in order to consider different environmental or operational influences in the data. Although whitening is a well-known transformation, the author is not aware of its wide application to this end. Damage localization can also be attempted if the changes in the data can be associated with a particular sensor.

In this paper, damage detection and localization are performed in the time domain. Time-domain and feature-domain methods for damage detection were compared [22], and it was found that the selected features were more sensitive to damage than the physical or virtual sensor data. This was probably due to the fact that the features had a higher SNR than the sensor data. However, damage detection in the time domain has certain advantages compared to that in the feature domain. For example, the data dimensionality is often lower and the number of data points larger, which is advantageous in statistical analysis. The algorithm can also be fully automated, as system identification is not necessary. In addition, each time history can be associated with a single sensor to localize damage. Therefore, if measurement error can be decreased, the performance of time-domain methods is expected to improve.

It should be noted that there are several techniques for damage detection and localization using vibration measurements. In addition to time domain or feature domain methods, the techniques can be categorized as physics-based or data-based approaches. The present paper is restricted to data-based methods so that no numerical model, for example, a finite element model, is needed.

It is by no means claimed that the proposed method is optimal, but a single algorithm was selected to study whether data compression and reconstruction can be successfully applied to detect and localize damage in the time domain. In fact, several techniques were compared to eliminate environmental or operational influences, resulting in very similar performance as whitening transformation. A number of statistical novelty detection methods were also tested, and the one with the most robust behavior was selected. A comparison of different techniques to detect and localize damage is out of the scope of this study. The user can actually choose any algorithm once the reconstructed data are available. Nevertheless, it is believed that the results presented in this paper are representative for many time-domain methods. Feature-domain methods are also beyond the scope of this paper, because many feature-extraction techniques already include noise reduction due to averaging and may not benefit from the proposed data compression and reconstruction. In fact, the reconstructed signals become correlated and are no longer independent. This may cause challenges in system identification.

The paper is organized as follows. Virtual sensing using Bayesian estimation is outlined in Section 2. Optimal sensor placement for data storage and reconstruction is also discussed. An algorithm for damage detection and localization follows in Section 3. In Section 4, the proposed method is studied with numerical simulations of ambient vibration measurements under variable environmental conditions. Concluding remarks are given in Section 5.

## 2. Data Compression and Reconstruction Using Bayesian Virtual Sensing

The objective is to store only a small percentage of the dense sensor network data so that the full data can be accurately reconstructed and used for damage detection. Different environmental conditions between measurements must be considered. It is assumed that the environmental conditions remain nearly constant during a single measurement. This can be justified because the measurement period is typically much shorter than the variations in the environment—for example, outdoor temperature.

The dynamic characteristics of the structure can change between measurements due to environmental or operational variability as well as due to damage. It is important to retain those variations during data compression and reconstruction. Therefore, each measurement is processed independently in this stage. Different measurements are pooled only in the damage-detection stage to distinguish between those two aforementioned influences.

**Figure 1:**
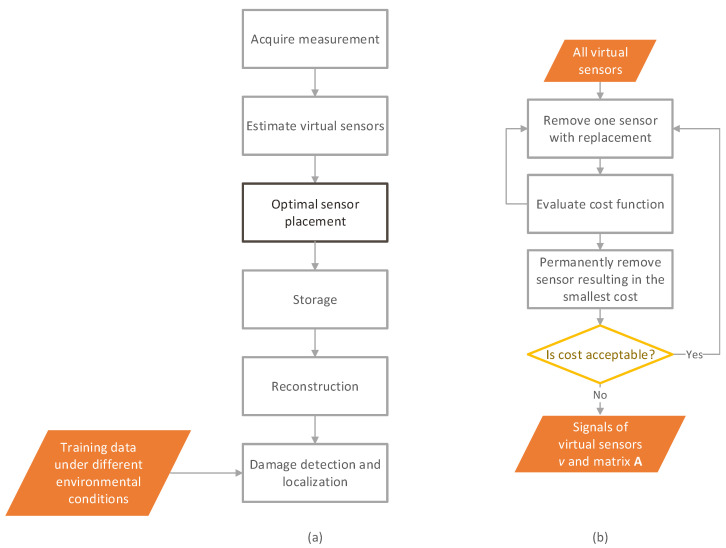
Flowcharts of (**a**) the whole process and (**b**) the optimal sensor placement (OSP) function.

Figure 1 shows the flowchart of the whole process from vibration measurement to damage identification. Each vibration measurement acquired with a sensor network is processed as follows. First, Bayesian virtual sensors [23] are designed to reduce the measurement error. Then, a limited number of virtual sensor signals are selected for storage based on the optimal sensor placement algorithm (Figure 1b). The excluded signals can be reconstructed using the stored signals and a coefficient matrix. The stored and reconstructed signals are finally used for damage detection and localization. To this end, training data from the undamaged structure under different environmental or operational conditions are used.

This section discusses data compression and reconstruction, while the next section covers damage detection and localization. The main theoretical novelty of this paper is included in Section 2.2, Section 2.3 and Section 2.4.

### 2.1. Empirical Bayesian Virtual Sensing

The objective of empirical virtual sensing is to decrease the measurement error of all sensors. The derivation of Bayesian virtual sensors [20] is briefly repeated for completeness. Consider a sensor network measuring *p* simultaneously sampled response variables $y=y\left(t\right)\in {\mathbb{R}}^{p}$ at time *t*. Each measured data point **y** includes measurement error $w=w\left(t\right)\in {\mathbb{R}}^{p}$:

(1) y=x+w,

where $x=x\left(t\right)\in {\mathbb{R}}^{p}$ are the exact values of the measured degrees of freedom. Equation (1) can be written in the following form at time *t* [24].

(2) $$\left\{\begin{array}{c} x\\ y \end{array}\right\}=\left[\begin{array}{cc} I & 0\\ I & I \end{array}\right]\left\{\begin{array}{c} x\\ w \end{array}\right\},$$

where **I** is the identity matrix of size *p* and **0** is the null matrix of size *p* × *p*. For simplicity but without loss of generality, assume zero-mean variables **x** and **y**. The partitioned covariance matrix is

(3) $$\left[\begin{array}{cc} \Sigma_{xx} & \Sigma_{xy}\\ \Sigma_{yx} & \Sigma_{yy} \end{array}\right]=E\left(\left\{\begin{array}{c} x\\ y \end{array}\right\}\left[\begin{array}{cc} x^{T} & y^{T} \end{array}\right]\right)=\left[\begin{array}{cc} I & 0\\ I & I \end{array}\right]E\left(\left\{\begin{array}{c} x\\ w \end{array}\right\}\left[\begin{array}{cc} x^{T} & w^{T} \end{array}\right]\right){\left[\begin{array}{cc} I & 0\\ I & I \end{array}\right]}^{T}=\left[\begin{array}{cc} I & 0\\ I & I \end{array}\right]\left[\begin{array}{cc} \Sigma_{xx} & 0\\ 0 & \Sigma_{ww} \end{array}\right]\left[\begin{array}{cc} I & I\\ 0 & I \end{array}\right]=\left[\begin{array}{cc} \Sigma_{xx} & \Sigma_{xx}\\ \Sigma_{xx} & \Sigma_{xx}+\Sigma_{ww} \end{array}\right]$$

where E( ) denotes the expectation operator and the measurement error **w** is assumed to be zero-mean Gaussian, independent of **x**, with a covariance matrix **Σ***_ww_*.

A linear minimum mean-square error (MMSE) estimate for **x**|**y** (**x** given **y**) is obtained by minimizing the mean-square error (MSE) [24]. The expected value, or the conditional mean, of the predicted variable is

(4) $$\hat{x}=E(x|y)=\Sigma_{xx}{\left(\Sigma_{xx}+\Sigma_{ww}\right)}^{-1}y=\Sigma_{xx}\Sigma_{yy}^{-1}y,$$

and the covariance matrix of the estimation error is

(5) $$\Sigma_{\mathrm{post}}=\mathrm{cov}(x|y)=\Sigma_{xx}-\Sigma_{xx}{\left(\Sigma_{xx}+\Sigma_{ww}\right)}^{-1}\Sigma_{xx}=\Sigma_{xx}-\Sigma_{xx}\Sigma_{yy}^{-1}\Sigma_{xx}.\;$$

It was shown that the virtual sensors (Equation (4)) are more accurate than the actual measurements [23].

### 2.2. Storing Physical Sensor Data

Let us assume that channels *v* of the physical measurement are stored. The objective is to reconstruct the signals of the whole sensor network. For simplicity but without loss of generality, assume zero-mean variables. The stored signals are the actual measurements **y***_v_*. Then, $E(x|y_{v})$ and $\mathrm{cov}(x|y_{v})$ are estimated using the partitioned mixed-data covariance matrix

(6) $$E\left(\left\{\begin{array}{c} x\\ y_{v} \end{array}\right\}\left[\begin{array}{cc} x^{T} & y_{v}^{T} \end{array}\right]\right)=\left[\begin{array}{cc} \Sigma_{xx} & \Sigma_{xx_{v}}\\ \Sigma_{x_{v}x} & \Sigma_{y_{v}y_{v}} \end{array}\right],$$

because $E\left(xy_{v}^{T}\right)=E\left(xx_{v}^{T}\right)$. The conditional mean (the reconstructed data) and the error covariance matrix are respectively obtained using MMSE:

(7) $$E(x|y_{v})=\Sigma_{xx_{v}}\Sigma_{y_{v}y_{v}}^{-1}y_{v}=By_{v}$$

and

(8) $$\mathrm{cov}(x|y_{v})=\Sigma_{xx}-\Sigma_{xx_{v}}\Sigma_{y_{v}y_{v}}^{-1}\Sigma_{x_{v}x},$$

where $B=\Sigma_{xx_{v}}\Sigma_{y_{v}y_{v}}^{-1}$ is the coefficient matrix that has to be stored along with the stored signals **y***_v_*.

### 2.3. Storing Virtual Sensor Data

Once a limited number of virtual sensor signals are stored, the omitted signals must be reconstructed. In the following, formulas for the reconstruction and its estimation error are derived.

Let us assume that the signals of channels *v* of the virtual sensors are stored, whereas the omitted signals of channels *u* must be reconstructed. The error variances of the stored signals are the diagonal elements of $\Sigma_{\mathrm{post},\mathrm{vv}}$, which is a submatrix of $\Sigma_{\mathrm{post}}$ (Equation (5)) consisting only rows *v* and columns *v*. When reconstructing the omitted signals using the stored virtual sensors’ data ${\hat{x}}_{v}$, the conditional mean $E(x_{u}|{\hat{x}}_{v})$ and covariance matrix $\mathrm{cov}(x_{u}|{\hat{x}}_{v})$ must be derived. The Bayesian virtual sensors are not exact, but follow the error model

(9) $$x=\hat{x}+e,$$

where **e** is the posterior error having a zero mean and covariance matrix $\Sigma_{\mathrm{post}}$ (Equation (5)). Thus,

(10) $$E(x_{u}|{\hat{x}}_{v})=E({\hat{x}}_{u}|{\hat{x}}_{v})=\Sigma_{{\hat{x}}_{u}{\hat{x}}_{v}}\Sigma_{{\hat{x}}_{v}{\hat{x}}_{v}}^{-1}{\hat{x}}_{v}=A{\hat{x}}_{v},$$

where $A=\Sigma_{{\hat{x}}_{u}{\hat{x}}_{v}}\Sigma_{{\hat{x}}_{v}{\hat{x}}_{v}}^{-1}$ is the coefficient matrix that has to be stored along with the stored signals ${\hat{x}}_{v}$. The data covariance matrix $\Sigma_{\hat{x}\hat{x}}$ is estimated using the full virtual sensor data. According to MMSE, the two terms in the right-hand side of Equation (9) are orthogonal [25]. Therefore, the covariances are related as

(11) $$\mathrm{cov}\left(x_{u}|{\hat{x}}_{v}\right)=\mathrm{cov}\left({\hat{x}}_{u}|{\hat{x}}_{v}\right)+\Sigma_{\mathrm{post},uu}=\Sigma_{{\hat{x}}_{u}{\hat{x}}_{u}}-\Sigma_{{\hat{x}}_{u}{\hat{x}}_{v}}\Sigma_{{\hat{x}}_{v}{\hat{x}}_{v}}^{-1}\Sigma_{{\hat{x}}_{v}{\hat{x}}_{u}}+\Sigma_{\mathrm{post},uu}.$$

The diagonal elements of this matrix are the variances of the reconstruction errors. Notice that the reconstruction error is higher than the error of the virtual sensor $\Sigma_{\mathrm{post},uu}$.

### 2.4. Comparison of the Two Storage Strategies

The question may arise as to whether the actual measurements **y***_v_* or the Bayesian estimates ${\hat{x}}_{v}$ (Equation (4)) of the selected channels *v* should be stored. The choice depends on the accuracy of the reconstruction. Intuitively, more accurate signals should be used for the reconstruction of signals of the sensors *u*. It is now proved that using ${\hat{x}}_{v}$ instead of **y***_v_* results in a smaller reconstruction error. The reconstructed signals are either $E(x_{u}|y_{v})$ or $E(x_{u}|{\hat{x}}_{v})$ in Equations (7) and (10), respectively. The corresponding error covariance matrices are given in Equations (8) and (11), respectively. In order to compare the error variances, some manipulation of Equation (11) is needed. Using Equation (4),

(12) $$\Sigma_{\hat{x}\hat{x}}=E\left(\hat{x}{\hat{x}}^{T}\right)=\Sigma_{xx}\Sigma_{yy}^{-1}E\left(yy^{T}\right)\Sigma_{yy}^{-1}\Sigma_{xx}=\Sigma_{xx}\Sigma_{yy}^{-1}\Sigma_{xx}.$$

Therefore,

(13) $$\Sigma_{{\hat{x}}_{u}{\hat{x}}_{u}}=\Sigma_{x_{u}x}\Sigma_{yy}^{-1}\Sigma_{xx_{u}}.$$

Substituting Equations (5) and (13) into Equation (11) results in

(14) $$\mathrm{cov}(x_{u}|{\hat{x}}_{v})=\mathrm{cov}({\hat{x}}_{u}|{\hat{x}}_{v})+\Sigma_{\mathrm{post},uu}=\left(\Sigma_{x_{u}x}\Sigma_{yy}^{-1}\Sigma_{xx_{u}}-\Sigma_{{\hat{x}}_{u}{\hat{x}}_{v}}\Sigma_{{\hat{x}}_{v}{\hat{x}}_{v}}^{-1}\Sigma_{{\hat{x}}_{v}{\hat{x}}_{u}}\right)+\;\left(\Sigma_{x_{u}x_{u}}-\Sigma_{x_{u}x}\Sigma_{yy}^{-1}\Sigma_{xx_{u}}\right)=\Sigma_{x_{u}x_{u}}-\Sigma_{{\hat{x}}_{u}{\hat{x}}_{v}}\Sigma_{{\hat{x}}_{v}{\hat{x}}_{v}}^{-1}\Sigma_{{\hat{x}}_{v}{\hat{x}}_{u}}.$$

This is compared with the error covariance $\mathrm{cov}(x_{u}|y_{v})$. Consider any single sensor in the set *u*. Then the covariance matrix becomes the variance. The difference is

(15) $$\begin{array}{c} \mathrm{cov}(x_{u}|y_{v})-\mathrm{cov}(x_{u}|{\hat{x}}_{v})\\ =\Sigma_{x_{u}x_{u}}-\Sigma_{x_{u}x_{v}}\Sigma_{y_{v}y_{v}}^{-1}\Sigma_{x_{v}x_{u}}-\left(\Sigma_{x_{u}x_{u}}-\Sigma_{{\hat{x}}_{u}{\hat{x}}_{v}}\Sigma_{{\hat{x}}_{v}{\hat{x}}_{v}}^{-1}\Sigma_{{\hat{x}}_{v}{\hat{x}}_{u}}\right)\\ =\Sigma_{x_{u}x_{v}}\left(\Sigma_{{\hat{x}}_{v}{\hat{x}}_{v}}^{-1}-\Sigma_{y_{v}y_{v}}^{-1}\right)\Sigma_{x_{v}x_{u}}\\ >\Sigma_{x_{u}x_{v}}\left(\Sigma_{y_{v}y_{v}}^{-1}-\Sigma_{y_{v}y_{v}}^{-1}\right)\Sigma_{x_{v}x_{u}}=0. \end{array}$$

The following facts were used in the derivation: (1) The Bayesian virtual sensors are more accurate than the physical sensors, (2) all the covariance matrices are positive definite, and (3) the cross-correlations are equal:

(16) $$\Sigma_{x_{u}x_{v}}=\Sigma_{{\hat{x}}_{u}{\hat{x}}_{v}}.$$

The proof of Equation (16) is derived using Equation (9):

(17) $$\begin{array}{c} \Sigma_{x_{u}x_{v}}=E\left(x_{u}x_{v}^{T}\right)=E\left[\left({\hat{x}}_{u}+e_{u}\right){\left({\hat{x}}_{v}+e_{v}\right)}^{T}\right]\\ =E\left({\hat{x}}_{u}{\hat{x}}_{v}^{T}\right)+E\left({\hat{x}}_{u}e_{v}^{T}\right)+E\left(e_{u}{\hat{x}}_{v}^{T}\right)+E\left(e_{u}e_{v}^{T}\right)\\ =\Sigma_{{\hat{x}}_{u}{\hat{x}}_{v}}, \end{array}$$

because the last three terms in the middle row are zero.

From Equation (15), it can be concluded that the stored Bayesian virtual sensors ${\hat{x}}_{v}$ instead of the corresponding raw measurements **y***_v_* result in a smaller reconstruction error and should be preferred for storage. This will also be shown in the numerical experiments.

### 2.5. Optimal Sensor Placement

The stored signals are selected using an optimal sensor placement algorithm (Figure 1b). It is an iterative procedure starting with an initial large sensor network including all measured degrees of freedom (DOF). Each virtual sensor in turn is removed with replacement, and the error variances of all reconstructed signals are computed, which are the diagonal terms of the error covariance matrix (Equation (11)). The cost function for these reduced sensor networks is evaluated. The minimum cost is found and the reduced sensor network corresponding to this minimum becomes the new candidate set for the next round. In other words, the removed sensor corresponding to this minimum cost is permanently discarded. The process is repeated until the desired number of sensors or the allowed error limit is reached. Finally, the data from the remaining virtual sensors are stored together with matrix **A** in Equation (10) for reconstruction of the discarded sensors.

The objective function *f* used in this study is

(18) $$f\left(k,i\right)=\left\{\sigma_{cr,i}-\sigma_{i}|\mathrm{sensor}\;k\;\mathrm{removed}\right\}$$

where σ*_cr,i_* is the critical reconstruction error of sensor *i* defined by the user, and *σ_i_* is the current reconstruction error of sensor *i*. Notice that each sensor can be given a different threshold. The cost function *R* is the negative objective function:

(19) $$R\left(k,i\right)=\left\{\sigma_{i}-\sigma_{cr,i}|\mathrm{sensor}\;k\;\mathrm{removed}\right\}$$

The decision rule is the minimax criterion—that is, sensor *k* that minimizes the worst-case loss can be permanently removed:

(20) $$\underset{k}{\mathrm{argmin}}\;\underset{i}{\max}R\left(k,i\right)$$

or using the objective function *f*,

(21) $$\underset{k}{\mathrm{argmax}}\;\underset{i}{\min}f\left(k,i\right)$$

**Figure 2:**
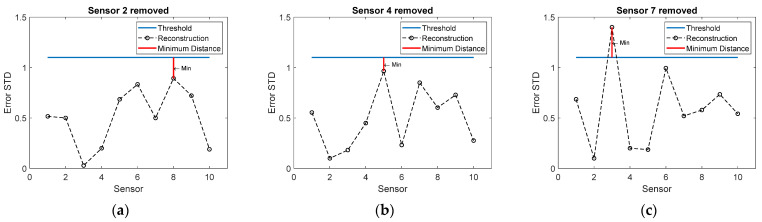
Reconstruction errors of each sensor when one sensor in turn out of sensors 2, 4, and 7 is removed. The remaining seven sensors were removed earlier and must be reconstructed. (**a**) Sensor 2 removed; (**b**) Sensor 4 removed; (**c**) Sensor 7 removed. The minimum distances from the threshold are also shown. The largest minimum distance from the threshold is in plot (**a**), indicating that sensor 2 can be permanently removed.

As an illustrative example, consider a sensor network with 10 sensors on a structure. Assume that the OSP algorithm has proceeded to the point where sensors 2, 4, and 7 are stored, whereas sensors 1, 3, 5, 6, 8, 9, and 10 must be reconstructed. The next round of the BSSP algorithm investigates whether one of sensors 2, 4, or 7 can be removed. To this end, sensor *k* = 2 is first removed, and all sensors except 4 and 7 are reconstructed. The estimated standard deviations of all sensors are plotted in Figure 2a. The errors of sensors 4 and 7 are posterior variances (Equation (5)), whereas the other errors are reconstruction errors (Equation (11)). The minimum distance from the threshold is found for sensor *i* = 8. The same procedure is performed by removing sensor *k* = 4 (Figure 2b) and sensor *k* = 7 (Figure 2c) and recording the minimum distances shown in the figures, from which the maximum is found (Figure 2a). The decision rule then says that sensor 2 can be permanently removed. By storing only sensors 4 and 7, the remaining signals can be reconstructed with tolerable error. The removal continues until all trials result in a similar case as in Figure 2c, where the error threshold is exceeded. In that case no more sensors can be removed and the procedure is terminated.

Notice that the threshold cannot be given an arbitrary low value, but it must be greater than the posterior variance for all sensors. This is because the reconstruction error is always greater than the posterior error.

## 3. Damage Detection and Localization

**Figure 3:**
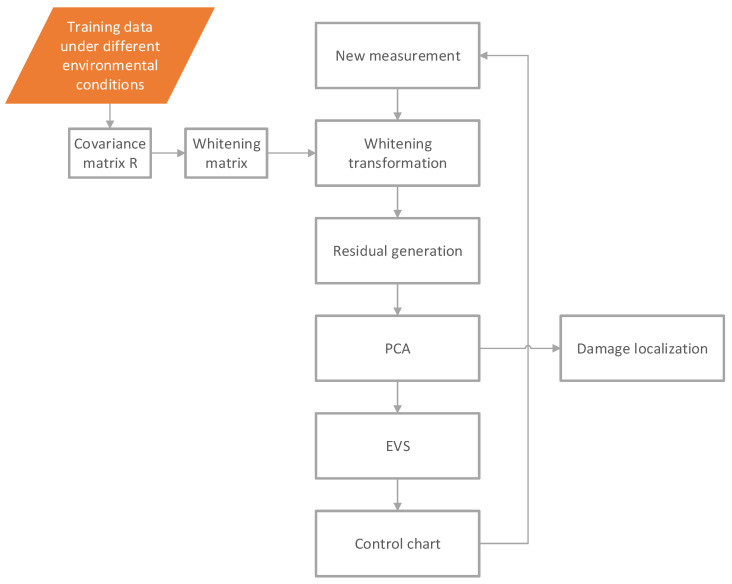
Flowchart of damage detection and localization.

Processing a single measurement yields both data compression and noise reduction. The next step is to use the full data (stored and reconstructed signals) of all measurements in damage detection. The flowchart for damage detection and localization is plotted in Figure 3. Notice that one possible algorithm is only introduced, but the user is free to apply any other method once the stored and reconstructed data are available.

The stored and reconstructed data are used for damage detection in the time domain. First, the mean vector and the covariance matrix are estimated using training data from the undamaged structure under different environmental or operational conditions. Whitening transformation is applied to the training data [26]. This transformation is then fixed and applied to the test data. The transformed data are subjected to principal component analysis (PCA). Retaining the first principal component only, the data dimensionality is decreased to one. An extreme value statistics (EVS) control chart is then designed for the first PC scores with appropriate control limits and subgroup size [20,27,28,29]. In this paper, the probability of false alarms equal to 0.001 was used.

Damage location is assumed to correspond to the direction of the first principal component of the residuals. The largest projection of the first PC on the sensor coordinates reveals the sensor closest to damage.

It is essential to model the data of each measurement independently for compression and reconstruction so that the environmental, operational, or damage effects are retained during this first phase. Elimination of the environmental or operational influences is performed in the second phase, in which several measurements are pooled to build a data model of the undamaged structure under different environmental or operational conditions. Novelty detection is then applied to the test data using the data model of the second phase. Each step is discussed in more detail in the following.

### 3.1. Spatial and Spatiotemporal Correlation

Covariance-based methods in vibration-based structural health monitoring are effective and quite common. The covariance matrix is estimated as follows. Simultaneous time series of the sensor network are formed by combining the stored and reconstructed signals. Training data are formed by pooling several measurements from the undamaged structure. If the process can be assumed stationary with zero mean, temporal correlation can also be utilized [30]. The time-shifted covariance matrix estimate, with a time shift *i*, is computed by

(22) $${\hat{R}}_{i}=\frac{1}{N-i}\overset{N-i}{\underset{k=1}{\sum }}\left[x\left(k\right)x{\left(k+i\right)}^{T}\right],$$

where $x\left(k\right)=x\left(k\Delta t\right)$ is the *k*th sample at a time instant kΔt where Δt is the sampling period and *N* is the number of samples. If *m* is the model order, the covariance matrix is

(23) $$R=\left[\begin{array}{cccc} R_{0} & R_{1} & \cdots & R_{m}\\ R_{1}^{T} & R_{0} & \cdots & R_{m-1}\\ \vdots & \vdots & \ddots & \vdots \\ R_{m}^{T} & R_{m-1}^{T} & \vdots & R_{0} \end{array}\right]$$

If spatial correlation is only studied, the correlation matrix is simply $R_{0}$. Spatial correlation is related to mode shapes, whereas temporal correlation also takes the frequency information into account. The covariance matrix is estimated using the training data.

### 3.2. Data Normalization Using Whitening Transformation

**Figure 4:**
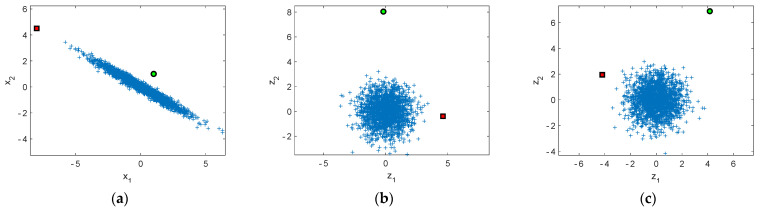
An illustrative example of whitening. (**a**) Original variables; (**b**) whitening transformation using **W**_1_; (**c**) whitening transformation using **W**_2_. The two isolated points represent outliers in the signal space (red square) and in the noise space (green circle).

Environmental or operational variability often affects the dynamic characteristics of the structure, which can mask the effects of damage. Fortunately, in a multivariate case, the variables are often highly correlated, which affects the covariance structure of the training data (see an example in Figure 4a). Once damage occurs, the covariance structure also changes. Consequently, the new data points are assumed to stand out like the two isolated data points in Figure 4.

Whitening transformation is applied to the training data to consider the environmental or operational influences without measuring the underlying quantities. The objective of whitening or “sphering” is to linearly transform the data vector into another vector such that the elements of the new vector are uncorrelated and have unit variances. The whitening transformation therefore results in a unit data covariance matrix. For example, let us have a two-dimensional variable with a multivariate Gaussian distribution (Figure 4a). After whitening, the distribution looks like a hypersphere (Figure 4b). The transformation is, however, not unique, as the axes in Figure 4b can be rotated, resulting in another possible transformation (Figure 4c). Two isolated data points, not included in the training data set, are highlighted to distinguish between these two different whitening transformations. The choice of transformation is important in damage localization, discussed in Section 3.7.

The symmetric data covariance matrix of the training data is first decomposed using spectral decomposition:

(24) $$R=E\left(xx^{T}\right)=UDU^{T},$$

where **D** is a diagonal matrix with eigenvalues, and **U** is an orthogonal matrix:

(25) $$U^{T}U=UU^{T}=I,$$

where **I** is the identity matrix.

Whitening is a linear transformation from the original variables **x** into **z**:

(26) $$z=W_{1}x,$$

in which the whitening matrix **W**_1_ is

(27) $$W_{1}=D^{-\frac{1}{2}}U^{T},$$

To show that Equation (26) is a whitening transformation, the covariance matrix of **z** should be the identity matrix:

(28) $$E\left(zz^{T}\right)=W_{1}E\left(xx^{T}\right)W_{1}^{T}=D^{-\frac{1}{2}}U^{T}UDU^{T}UD^{-\frac{1}{2}}=I$$

Whitening does not result in dimensionality reduction, and further processing is typically needed.

Notice how whitening transformation differs from the subspace methods that project the data onto the signal space and the noise space. As an example, Figure 4a illustrates two points, from which the red square lies in the signal space and the green circle in the noise space. Subspace methods can only detect damage in the noise space, whereas a data point in the signal space remains undetected. The whitening transformation instead does not divide the data into two subspaces, which can in some cases be advantageous. In other cases, it may not be desirable to have alarms when the data points lie in the signal space, e.g., in cases where extreme temperature causes the data to fall outside the training data. In the present example, the user has to decide whether the red square in Figure 4a is an indication of damage or merely an extreme environmental or operational condition.

### 3.3. Residual Generation

The variable **z** in Equation (26) can serve as a residual without further processing. It has a unit covariance matrix in the undamaged case. The correlation structure is expected to change due to damage because of changes in the mode shapes (spatial correlation) or additionally in the natural frequencies (spatiotemporal correlation). If the structure is damaged, the whitening transformation (Equation (26)) does not necessarily transform the new data points inside the hypersphere. Instead, it is expected that the transformed data points are located further away from the center of the hypersphere (see Figure 4). If the distance from the center is statistically significant, it is an indication of damage.

### 3.4. Principal Component Analysis

Whitening transformation of the training data results in data points that fall inside a hypersphere with a unit covariance matrix. Once damage occurs, it is expected that the new data points are located outside this hypersphere after the same transformation. It is also probable that the new data points are located in a certain direction from the center of the hypersphere. This direction can be found using principal component analysis (PCA) [4]. Therefore, PCA is applied to all of the data, both training and test data. The first principal component (PC) is only chosen for dimensionality reduction, which is expected to remove the curse of dimensionality. The PC scores of the first PC are used for damage detection and the first PC for damage localization.

### 3.5. Extreme Value Statistics

After principal component analysis, the dimensionality of the residuals is reduced to one. The distribution of this one-dimensional feature is not known, or more specifically, normal distribution may not be assumed. To this end, an extreme value distribution can be utilized [27]. The maxima or minima of a large set of independent, identically distributed random variables can be modelled with a generalized extreme value (GEV) distribution [28]. The data are divided into subgroups of *n* data points. The minimum and maximum from each batch are recorded, resulting in data called block minima and block maxima. The parameters of the two GEV distributions are identified for the block minima and block maxima of the data from the undamaged structure [20].

### 3.6. Control Chart

Control charts, designed for the extreme values, are used for novelty detection with the control limits computed according to the in-control data from the undamaged structure [29]. The control limits are computed from the GEV distributions by choosing the probability of exceedance (here 0.001). If the plotted new data points exceed the control limits, it is a possible indication of damage.

### 3.7. Damage Localization

As mentioned before, the whitening matrix (Equation (27)) is not unique. Multiplication with an orthogonal matrix also results in a whitening transformation [21]. A special whitening matrix is the inverse square root of **R**:

(29) $$W_{2}=UD^{-\frac{1}{2}}U^{T},$$

which is advantageous in damage localization, as it preserves the orientation in the original variable space (Figure 4c). The transformed variable with the largest value in the first principal component is assumed to reveal the sensor closest to damage.

Notice that for damage detection, it makes no difference which whitening matrix is used.

## 4. Numerical Experiment

**Figure 5:**
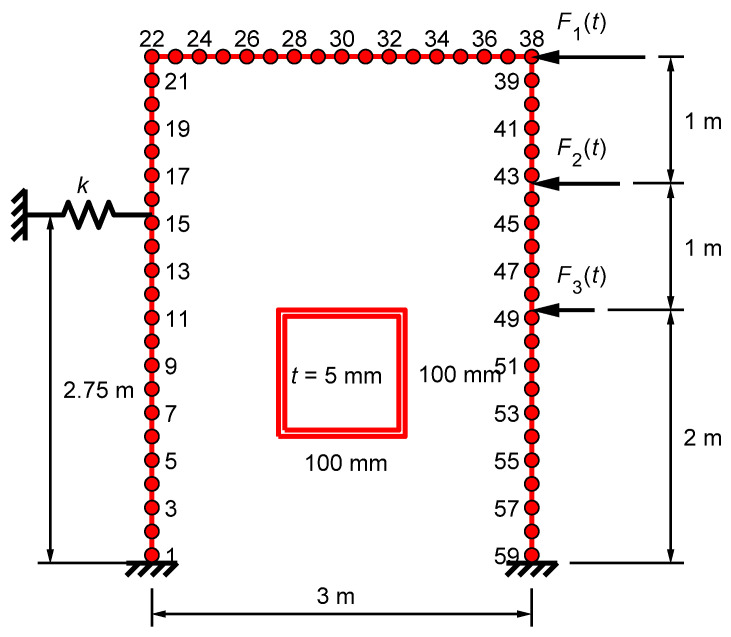
Frame structure with 59 accelerometers. Every other sensor number is displayed. Strain sensors were located almost at the same points. The intact cross-section is also shown.

**Figure 6:**
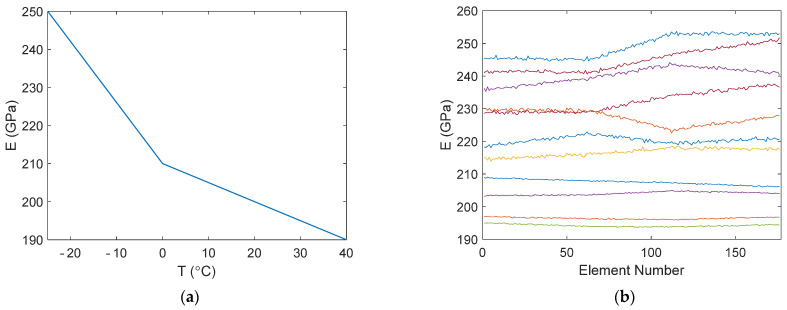
(**a**) Young’s modulus versus temperature; (**b**) sample distributions of the Young’s modulus.

Damage detection and localization were studied using simulated data. A two-dimensional finite element model of a steel frame with an additional spring element was subjected to three uncorrelated random excitations (Figure 5). The density of the steel was *ρ* = 7850 kg/m^3^ and the spring constant was *k* = 2.0 MN/m. The relationship between temperature and the Young’s modulus of steel was stepwise linear, as shown in Figure 6a. The frame was modelled with 176 simple beam elements, with a cross-section shown in Figure 5. The first seven modes were used in the simulation. Modal damping was assumed with damping ratios of *ζ*_1–2_ = 0.01, *ζ*_3_ = 0.015, and *ζ*_4–7_ = 0.02.

The three loads in the simulations were pseudorandom periodic excitations in the frequency range between 0 and 53.33 Hz with random amplitudes and phases [5]. All analyses had different loading functions. Steady-state analyses were performed in the frequency domain using modal superposition. Lateral accelerations at 59 points (every third node) and strains in the middle of 59 beam elements (every third element) were recorded (Figure 5). The measurement period was 32.77 s with a sampling frequency of 250 Hz. Each measurement consisted of 8192 samples per channel. Independent and identically distributed Gaussian random noise was added to each sensor. The average SNR was 30 dB for accelerometers and 10 dB for strain gauges. The standard deviations of the noise in all sensors were assumed to be known. They can also be approximated from the measurement data [23].

A rather complex but also fairly realistic temperature distribution was assumed. The temperature of the upper left (*UL*) corner, *T_UL_*, varied randomly between −25 °C and +40 °C. The temperature at the upper right (*UR*) corner was *T_UR_* = *T_UL_* ± 5 °C, at the bottom left (*BL*) support was *T_BL_* = *T_UL_* ± 3 °C, and at the bottom right (*BR*) support was *T_BR_* = *T_UR_* ± 3 °C. Temperature variation between the aforementioned points was assumed to be linear, except that Gaussian random noise with a standard deviation of 0.2 °C was added to each element. Temperature within a single element was constant. Sample distributions of the Young’s modulus in the elements are plotted in Figure 6b. The distribution did not change during a single measurement.

**Figure 7:**
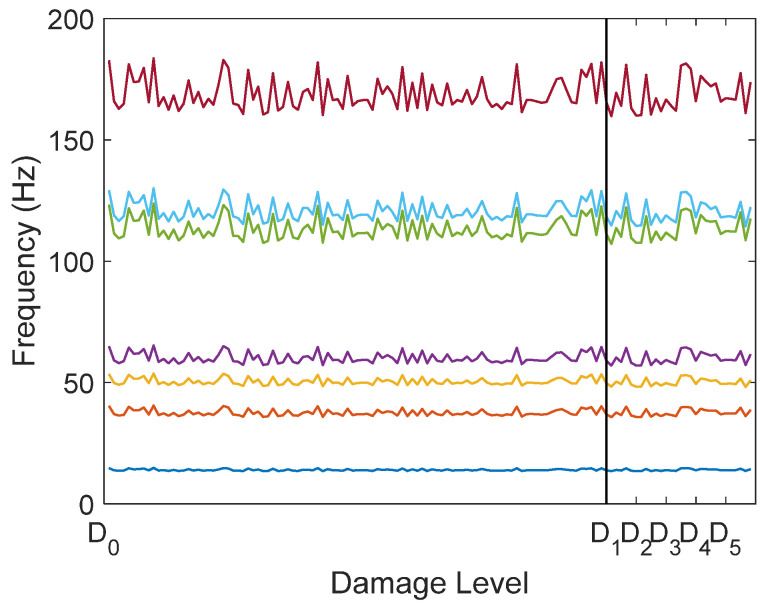
Variation of the seven lowest natural frequencies due to temperature and damage. Frequencies on the right of the vertical line are from the damaged structure. Different damage levels are also indicated.

The variations of the lowest natural frequencies between measurements due to temperature or damage are shown in Figure 7. A measurement with incipient damage is indicated with a vertical line. The natural frequencies were not used for data analysis, and were only plotted to illustrate how damage was masked by the temperature influence.

If modal parameters were used for damage identification, the following considerations should be made. Only three natural frequencies exist in the frequency range of the excitation. Three features are not enough to remove the environmental effects. If mode shapes were also used, the resulting dimensionality of the feature vector would be 3 + 3 (59 − 1) = 177 for real modes and 3 + 3 (59 − 1) 2 = 351 for complex modes (−1 is due to the scaling of the mode shape vectors). These large numbers would probably result in the curse of dimensionality, because one measurement yields only a single data point in the high-dimensional space. Some other issues in system identification were already mentioned in the introduction.

Visually unobservable damage at the support of the left leg was modelled with the removal of material inside the cross-section of the bottom element. The length of the element was 62.5 mm. Five different damage levels were studied with wall thicknesses of 4.5, 4.0, 3.5, 3.0, and 2.5 mm (Table 1). Notice that as material was removed, both the stiffness and mass decreased.

The first 100 measurements were acquired from the undamaged structure and each damage level was monitored with six measurements under random and unknown environmental conditions. Training data were the first 70 measurements. The extreme value statistics (EVS) control charts were designed using the same training data.

### 4.1. Bayesian Virtual Sensing and Selection of Sensors for Storage

**Figure 8:**
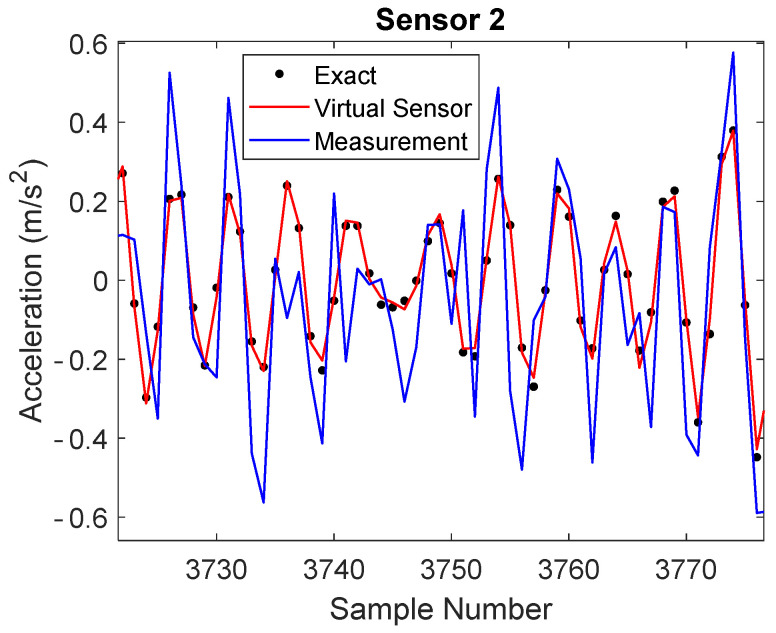
Detail of time history of accelerometer 2 in measurement 1 (undamaged): measured data, virtual sensor data, and noiseless data.

Each vibration measurement was individually processed, as illustrated in Figure 1. Bayesian virtual sensing resulted in noise reduction. A detail of the measured and estimated accelerations of sensor 2 in measurement 1 (undamaged) is plotted in Figure 8. The exact values are also shown. It can be seen that the Bayesian virtual sensor was more accurate than the corresponding physical sensor. The same applied to the other sensors.

**Figure 9:**
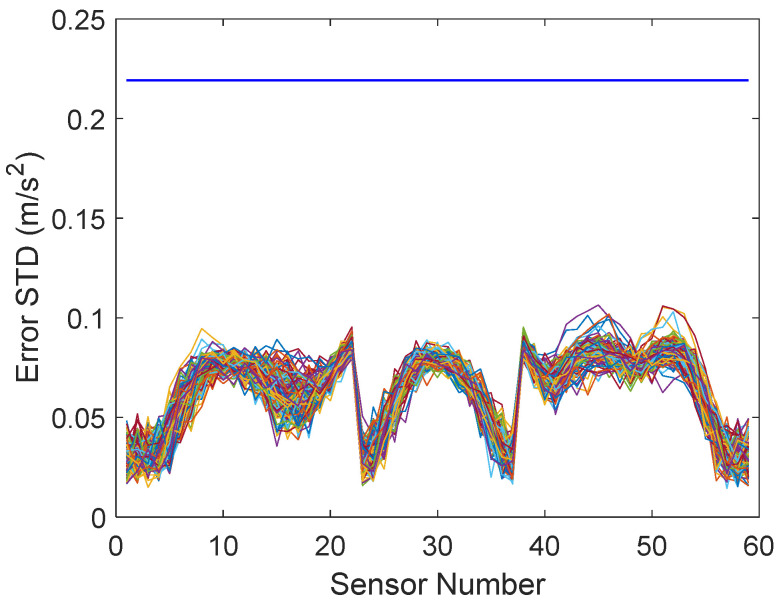
Measurement errors (blue horizontal line) and Bayesian virtual sensor errors of all sensors in each measurement (all damage levels).

The estimation errors of each virtual sensor in all measurements are plotted in Figure 9. The variability between measurements was quite small. Although the measurement errors were equal (blue horizontal line), the virtual sensor errors differed between sensors. All virtual sensors were more accurate than the hardware.

Next, a subset of the Bayesian virtual sensors was selected separately for each measurement by applying the backward sequential sensor placement (BSSP) approach [17]. The requirement was that the standard deviation of the measurement error had to be decreased at least 50% in all sensors.

The negative cost function was the minimum difference between the allowed and current reconstruction errors in any sensor in the network. The reduced network with the maximum distance (minimum cost) was selected for the next round. As a result, a single sensor was permanently removed from the network. Sensor removal continued until the accuracy criterion was violated. The required number of virtual sensors was five for most measurements.

**Figure 10:**
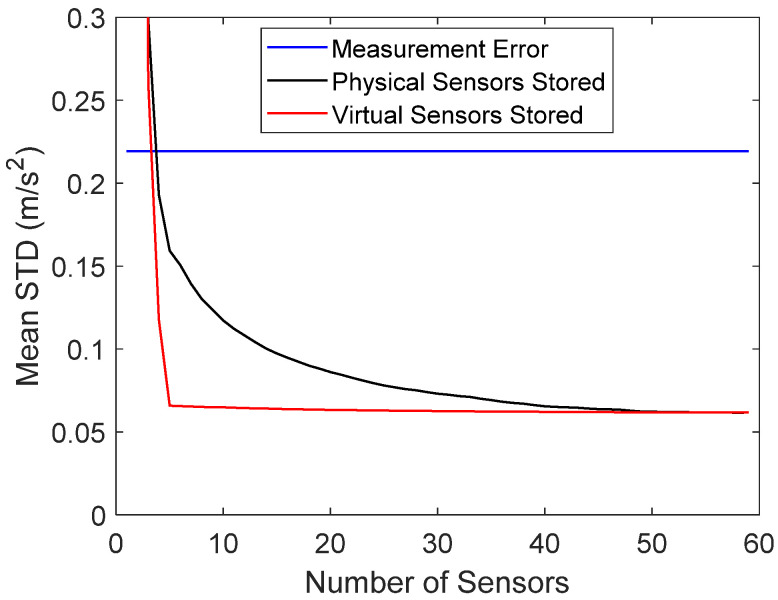
Mean reconstruction error as a function of the number of stored physical or virtual sensor signals in measurement 1. The blue horizontal line is the measurement error.

Once a single sensor was permanently removed, the errors of the stored and reconstructed virtual sensors were evaluated. The mean error of the full data as a function of the number of stored sensors is plotted in Figure 10 for measurement 1 (undamaged). It can be seen that storing only five virtual sensors instead of all 59 virtual sensors did not significantly increase the average noise level. When the number of stored sensors was further decreased below five, the reconstruction error increased considerably. On the other hand, when raw data were stored, the mean reconstruction error was larger. This was also theoretically proven (see Equation (15)).

**Figure 11:**
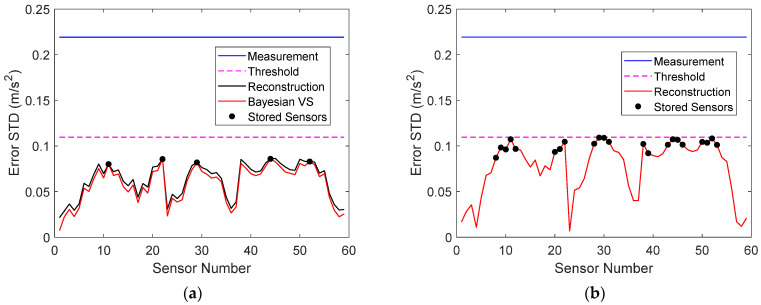
(**a**) Measurement error, virtual sensor error, and reconstruction error of all sensors in measurement 1, when five virtual sensor signals were stored. (**b**) Measurement error and reconstruction error of all sensors in measurement 1, when 22 physical sensor signals were stored. The allowable error is also shown. The stored sensors are indicated with dots.

The standard deviations of the measurement error, Bayesian virtual sensor error, and reconstruction error of all sensors in measurement 1 are plotted in Figure 11a. It can be seen that the reconstruction error was only slightly larger than that in the Bayesian virtual sensors. Sensors, for which the two errors were equal, corresponded to the stored signals, which were not reconstructed. They are indicated with black dots. The allowable error is also shown. The reconstruction errors were clearly smaller than requested. When raw data were stored, 22 signals were needed for reconstruction (Figure 11b), and for the most part, the reconstruction errors were larger than when five virtual sensor signals were stored.

**Figure 12:**
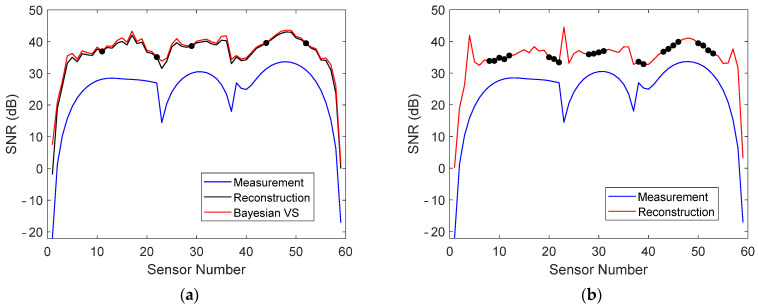
(**a**) SNR of physical sensors, Bayesian virtual sensors, and stored and reconstructed virtual sensors in measurement 1 when five virtual sensor signals were stored. (**b**) SNR of physical sensors and stored and reconstructed sensors in measurement 1 when 22 physical sensor signals were stored. The stored sensors are indicated with dots.

The reconstruction errors of sensors near the supports were small. The reconstruction error is, however, not the only important quantity. A high signal-to-noise ratio (SNR) is crucial for detection [18]. The SNR of the stored and reconstructed data in measurement 1 is plotted in Figure 12a when five virtual sensors were stored, as well as the SNR of the reconstruction when 22 physical sensors were stored. It can be seen that the sensors near the supports actually had a very low SNR, which could have an adverse effect on damage detection and localization. Nevertheless, the SNR of each stored and reconstructed virtual sensor was larger than that of the corresponding physical sensor and, for the most part, was also larger than the SNR when 22 physical sensors were stored.

**Figure 13:**
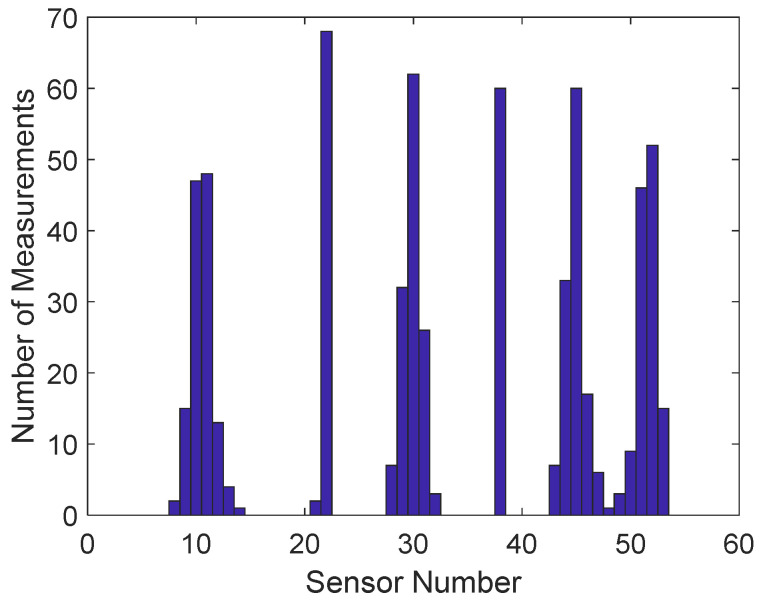
Histogram of selected virtual sensors for storage in all measurements (all damage levels).

**Figure 14:**
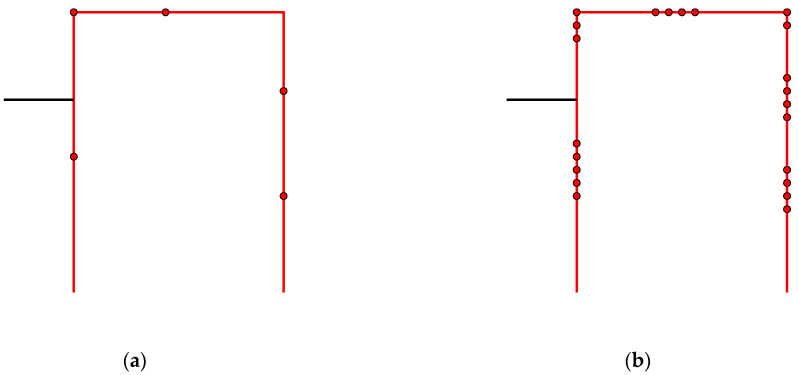
Selected sensors for storage in measurement 1: (**a**) virtual sensors 11, 22, 29, 44, and 52; (**b**) 22 physical sensors.

A histogram of the selected virtual sensors for storage in all measurements is shown in Figure 13. The most often selected sensors were located in six different regions of the structure. The placement of the stored virtual and physical sensors in measurement 1 is plotted in Figure 14a,b, respectively. Notice that no sensors were selected close to damage location (sensor 1).

The data compression ratio was computed as follows. If all data were stored, the number of floating-point numbers in each measurement was 59 × 8192 = 483,328 numbers. Storing five virtual sensor signals and the coefficient matrix **A** (Equation (10)) of size 54 × 5 resulted in 41,230 numbers. Consequently, only 8.5% of the total data had to be stored.

If raw signals were stored with the same requirement for the reconstruction accuracy, a larger number of signals had to be stored and all signals had to be reconstructed. Therefore, storing 22 signals and the coefficient matrix **B** (Equation (7)) of size 59 × 22 resulted in 181,522 numbers. Consequently, 37.6% of the total data had to be stored and the accuracy was still lower that when five virtual sensors were stored, as seen in Figure 11.

### 4.2. Damage Detection and Localization Using Spatial Correlation

**Figure 15:**
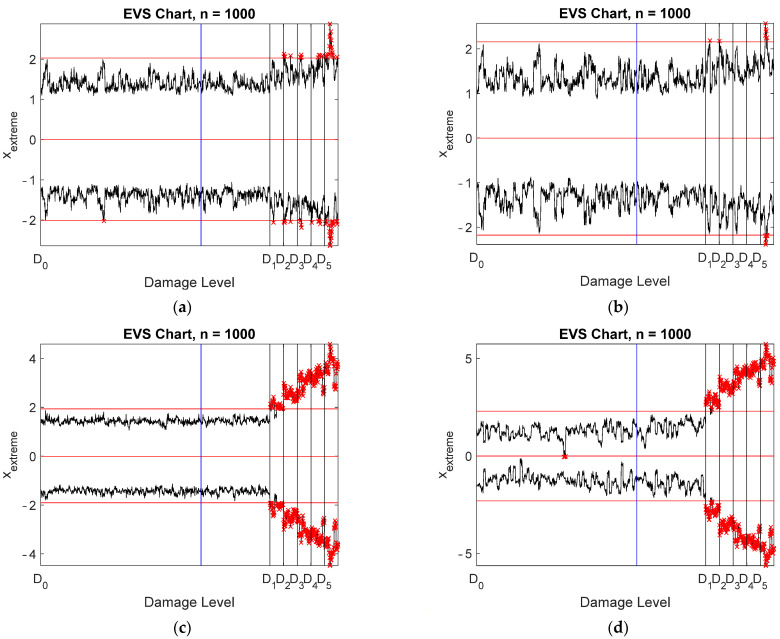
Damage detection using EVS control charts with spatial correlation: (**a**) all physical sensors; (**b**) seven virtual sensors: 10, 11, 22, 30, 38, 45, and 52; (**c**) all Bayesian virtual sensors; (**d**) stored and reconstructed virtual sensors. The vertical lines correspond to the end of training data (blue) and the five damage levels (black).

Damage detection was studied using four different data: raw measurements, all virtual sensors without compression, stored and reconstructed signals, and stored signals only. Spatial correlation was applied. EVS control charts were designed with a subgroup size of 1000 and are plotted in Figure 15. Notice that logarithmic scaling was applied to the vertical axis for clarity. The data points to the left of the blue vertical line correspond to the training data, whereas the black vertical lines indicate the onsets of the five damage scenarios. Only the largest damage level was clearly detected using the actual measurement data (Figure 15a). Classification of occasional out-of-control samples was difficult. All damage cases were detected using all virtual sensors (Figure 15c) or the stored and reconstructed data (Figure 15d). There is a slight difference between the two control charts, showing that the detection performance increased due to compression. This was quite a surprise, because the noise level in the reconstructed data was slightly larger than in the Bayesian virtual sensor data. The reason for this behavior is not known, and it is questionable whether this result can be generalized.

It may be argued that due to redundancy, only the selected virtual sensors would be enough for damage detection. This argument was tested by storing the seven most selected virtual sensors (sensors 10, 11, 22, 30, 38, 45, and 52; see Figure 13) from each measurement and designing an EVS control chart for these data (Figure 15b). Only the largest damage level was detected. Due to different environmental conditions between measurements, more than seven signals would have been needed to remove the environmental influences.

**Figure 16:**
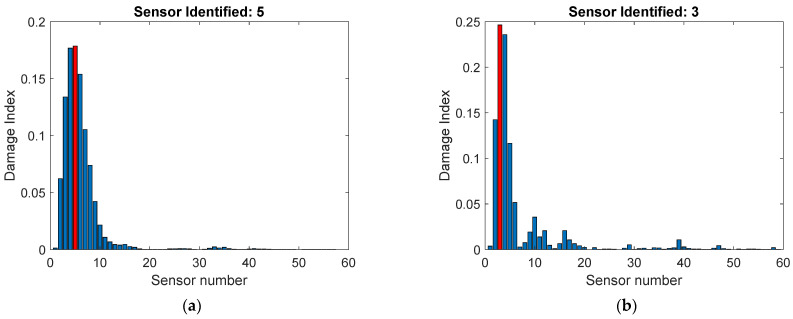
Damage localization: (**a**) all physical sensors; (**b**) stored and reconstructed virtual sensors. The correct damage position was closest to sensor 1.

Damage localization was done by plotting the squared projection of the first principal component on each sensor (Figure 16). Using the actual measurement data, damage was localized to sensor 5, and using the stored and reconstructed virtual sensors, damage was localized to sensor 3. Notice that sensor 3 was not included in the stored sensors, but its data were reconstructed. The correct position was closest to sensor 1. Neither analysis pointed to the correct sensor, but in either case, the suggested damage location was in the vicinity of the actual damage. The localization accuracy was slightly higher when the stored and reconstructed virtual sensors were used. The SNR in sensor 1 was very small, which probably resulted in the inaccuracy in damage localization. In many structures, damage may be located close to the fixed support, where the stresses are large but the vibration amplitude is very small, resulting in a small SNR. Therefore, strain measurements at these locations could be considered.

### 4.3. Damage Detection Using Spatiotemporal Correlation

Damage detection was also investigated with a spatiotemporal correlation model. The data and the algorithm remained the same. A model order equal to 30 was used. The size of the data matrix including the shifted data was very large, and a recursive algorithm was necessary to estimate the covariance matrices [31]. Covariance matrix estimates were needed in two stages: first, the covariance matrix of the training data for whitening, and second, the covariance matrix of all transformed data (residuals) for PCA.

**Figure 17:**
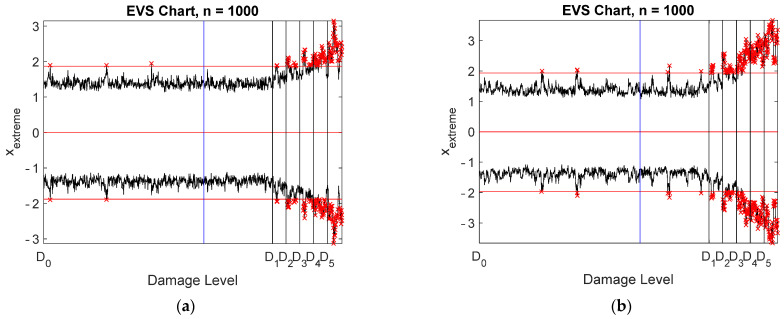

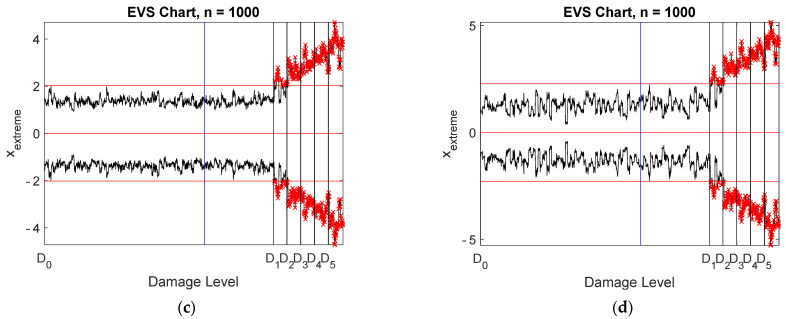
Damage detection using EVS control charts with a model order equal to 30: (**a**) all physical sensors; (**b**) seven virtual sensors: 10, 11, 22, 30, 38, 45, and 52; (**c**) all Bayesian virtual sensors; (**d**) stored and reconstructed virtual sensors. The vertical lines correspond to the end of training data (blue) and the five damage levels (black).

Spatiotemporal correlation models yielded EVS control charts, shown in Figure 17, which can be compared with the corresponding charts in Figure 15. Considerable improvement resulted with the raw measurement data (Figure 17a). Almost all damage levels could be detected. However, occasional false alarms emerged. Drastic improvement occurred with the stored signals of the selected seven virtual sensors (Figure 17b). Nearly all damage cases were detected, with occasional false indications of damage. Spatiotemporal correlation was necessary to make the data redundant. Almost no effect was observed with the virtual sensors (Figure 17c) or the stored and reconstructed virtual sensors (Figure 17d). The detection performance slightly decreased, but was nevertheless almost perfect.

### 4.4. Strain Measurements

**Figure 18:**
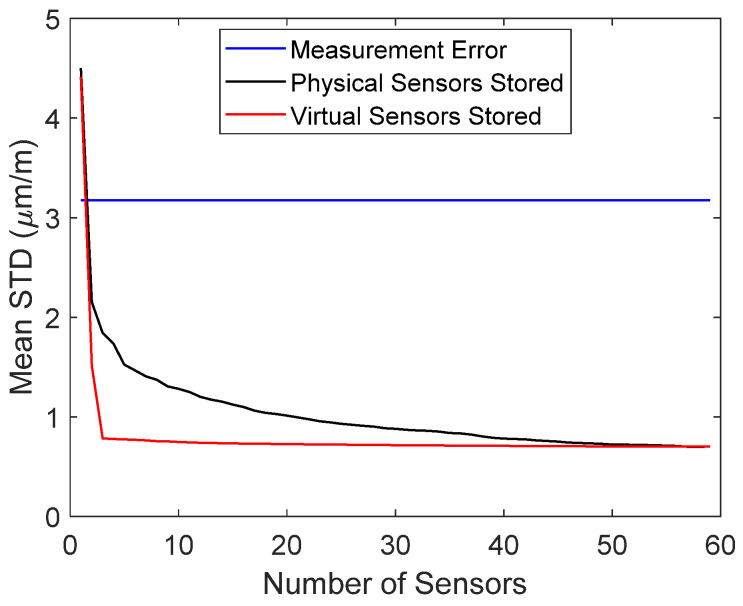
Mean reconstruction error as a function of the number of stored physical or virtual sensor signals in measurement 1. The blue horizontal line is the measurement error.

**Figure 19:**
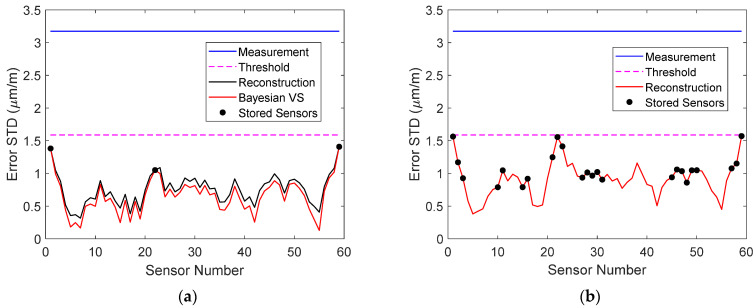
(**a**) Measurement error, virtual sensor error, and reconstruction error of all sensors in measurement 1 when virtual sensor signals were stored. (**b**) Measurement error and reconstruction error of all sensors in measurement 1 when physical sensor signals were stored. The allowable error is also shown. The stored sensors are indicated with dots.

**Figure 20:**
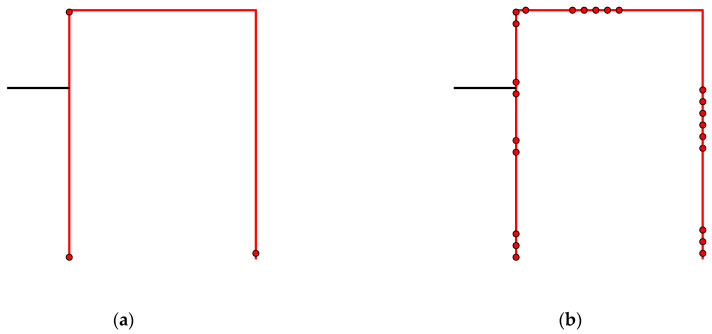
(**a**) Selected virtual sensors for storage in measurement 1: sensors 1, 22, and 59. (**b**) A total of 24 selected physical sensors for storage in measurement 1.

**Figure 21:**
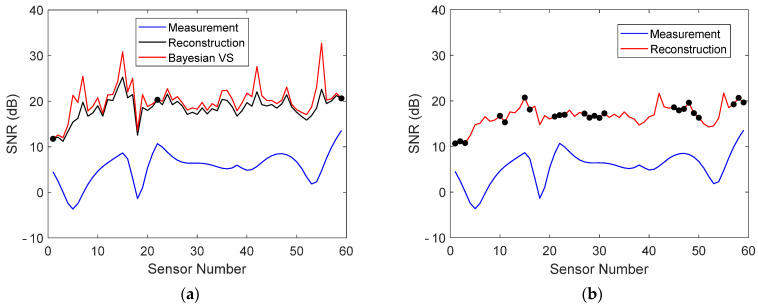
(**a**) SNR of physical sensors, Bayesian virtual sensors, and stored and reconstructed virtual sensors in measurement 1 when three virtual sensor signals were stored. (**b**) SNR of physical sensors and stored and reconstructed virtual sensors in measurement 1 when 24 physical sensor signals were stored. The stored sensors are indicated with dots.

**Figure 22:**
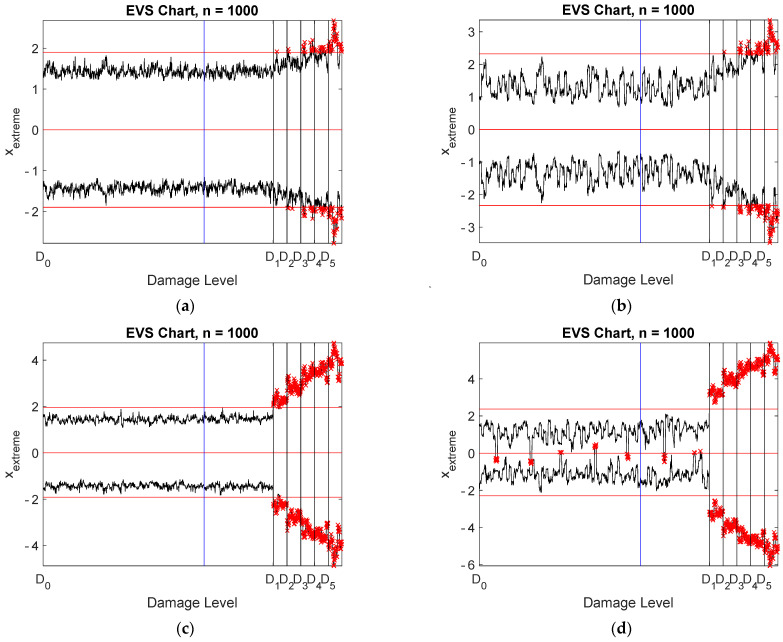
Damage detection using EVS control charts with spatial correlation: (**a**) all physical sensors; (**b**) seven virtual sensors: 1, 22, 31, 32, 38, 46, and 59; (**c**) all Bayesian virtual sensors; (**d**) stored and reconstructed virtual sensors. The vertical lines correspond to the end of training data (blue) and the five damage levels (black).

Strain measurements yielded very similar results and conclusions as the acceleration measurements. The main observations are briefly discussed. The most important result was that a strain sensor must be located very close to damage. Otherwise, detection may not be possible. Only three virtual strain sensors were selected for storage from most measurements (Figure 18). The number of physical sensors was considerably larger. The accuracy requirement was satisfied with just three virtual sensors (Figure 19a) or with 24 physical sensors (Figure 19b). The virtual sensors were located at the supports and at the left corner of the frame (Figure 20a), whereas the positions of the stored physical sensors are shown in Figure 20b. The SNRs of the virtual strain sensors were more uniform than those of the accelerometers (Figure 21). Spatial correlation analyses yielded the control charts shown in Figure 22. Notice that seven stored signals were used for Figure 22b, whereas only three signals were stored for Figure 22d. Three damage levels were detected using the raw data (Figure 22a) or seven selected virtual sensors (Figure 22b). All damage levels were detected using all virtual sensors (Figure 22c) or the stored and reconstructed virtual sensors (Figure 22d). The out-of-control samples in Figure 22d, in which the block maximum is negative or the block minimum is positive, can be ignored. Again, quite surprisingly, the stored and reconstructed virtual sensors yielded a slightly better detection performance than when all virtual sensors were stored. Damage was correctly localized to sensor 1. When sensor 1 was removed from the measurements, the detection performance decreased drastically.

Storing three virtual sensor signals and the coefficient matrix **A** (Equation (10)) of size 56 × 3 resulted in 24,744 numbers. Consequently, only 5.1% of the total data had to be stored.

### 4.5. Different Damage Locations

**Figure 23:**
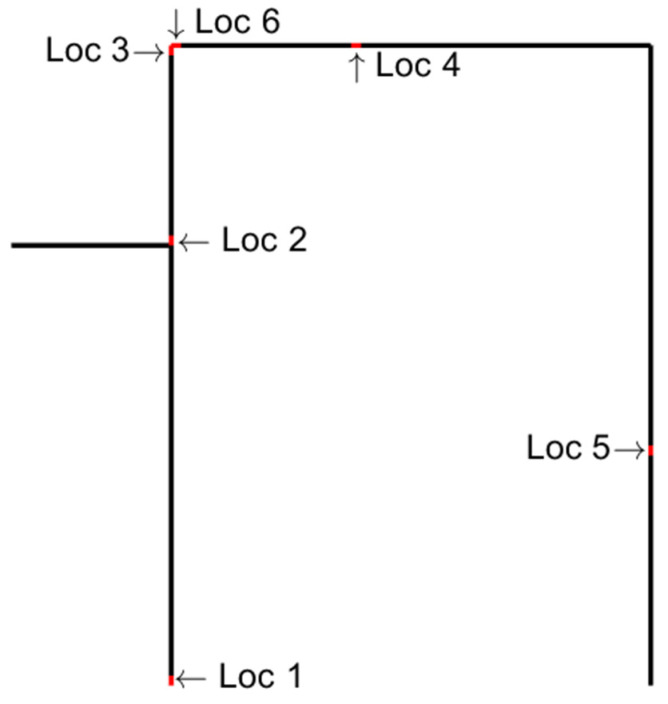
Six damage locations.

Damage detection and localization were also studied with different locations of damage. The same damage type and levels were assumed, as shown in Table 1. Both acceleration and strain measurements were considered. Six damage locations were arbitrarily selected, including the location presented earlier. They are plotted in Figure 23.

The results of damage detection and localization are given in Table 2, Table 3 and Table 4 for raw data, virtual sensor data, and stored and reconstructed virtual sensor data, respectively. Very interesting observations can be made: (1) Virtual sensors outperformed raw data in damage detection. (2) Damage detection performance was better when using stored and reconstructed virtual sensor data than all virtual sensors. (3) Damage localization performance was affected by the damage position and the sensor type. Either accelerometers or strain sensors, but not both, were able to localize damage in a certain position. This was especially true with the stored and reconstructed virtual sensor data (Table 4). (4) Detection of damage at location 2 was difficult with accelerometers. (5) Detection of minor damage at locations 2 and 5 was challenging using strain sensors.

### 4.6. Different Damage Detection Algorithms

**Figure 24:**
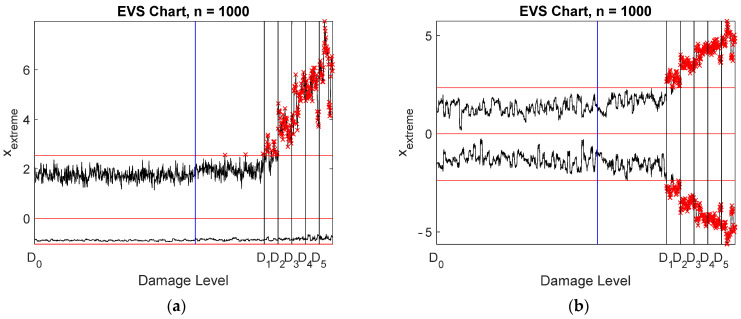
Damage detection from the stored and reconstructed virtual acceleration signals using two alternative algorithms and EVS control charts. (**a**) MD; (**b**) MMSE. The vertical lines correspond to the end of training data (blue) and the five damage levels (black).

As mentioned in the introduction, comparison of different damage detection algorithms is out of the scope of this study. If restricted to data-based methods in the time domain, two alternative methods were tested with the same data. Damage location 1 was assumed (Figure 23) to have the damage severities shown in Table 1. The two algorithms were Mahalanobis distance (MD) [32] and a regression-based minimum mean-square error (MMSE) estimation method [20]. MMSE was also applied to damage localization. Damage detection using the stored and reconstructed virtual accelerations is shown in Figure 24. Both methods were capable of detecting all damage levels. For the other data, the results are shown in Table 5. They can be compared with the results of the whitening algorithm shown in the first rows of Table 2, Table 3 and Table 4. MMSE performed slightly better than MD. Whitening outperformed the two alternative methods, but only slightly. Especially with the raw strain data, whitening could detect smaller damage than the other two algorithms.

## 5. Conclusions

A data-compression technique for storing and reconstructing simultaneously measured vibration signals in a dense sensor network was proposed. The stored and reconstructed data were used for damage detection and localization. Data compression and reconstruction for SHM is the main novelty of this paper.

The first step was to reduce measurement error by applying Bayesian virtual sensing. The virtual sensors, being more accurate than the physical sensors, replaced the actual measurements in the subsequent steps. The covariance matrix of the measurement errors was assumed to be diagonal and known. The measurement errors can also be different in each sensor, and they can be approximated from the measurement data. However, it is more difficult to estimate cross-correlated noise (full covariance matrix).

Data compression and reconstruction was done individually for each measurement, because the dynamic characteristics of the structure could vary between measurements due to environmental or operational variability, or damage. On the other hand, a full set of training data from several measurements under different environmental or operational conditions was used to build a covariance model of the undamaged structure. This model was applied to novelty detection using whitening transformation and principal component analysis. The first principal component was assumed to reveal the sensor closest to the damage location.

Data analysis for damage detection was performed in the time domain. No mathematical model of the structure nor system identification was needed. Spatial and spatiotemporal correlation models were compared. Spatiotemporal correlation gave no improvement over spatial correlation when all virtual sensors or stored and reconstructed virtual sensors were used. When raw measurement data or only the stored virtual sensor data were used, spatiotemporal correlation considerably increased the sensitivity to damage. A strain sensor had to be located close to damage. Otherwise, damage remained undetected. Accelerometers were also able to detect remote damage.

The main results are (1) it is more beneficial to store virtual sensor data than physical sensor data. (2) Less than 8.5% of the total amount of virtual sensor data had to be stored in the studied example, whereas 37.6% of the physical sensor data had to be stored for the same accuracy. (3) The stored and reconstructed virtual sensor data were more accurate than the actual measurements. (4) The accuracy of the reconstructed virtual sensors was only slightly smaller than that of the Bayesian virtual sensors. (5) The errors of the Bayesian virtual sensors and, consequently, the reconstruction errors were not the same even when the measurement errors were equal. (6) Whitening transformation was able to take the environmental or operational influences into account without measuring the underlying variables. (7) Damage detection and localization were more reliable with the stored and reconstructed virtual sensors than with the actual measurements. (8) Damage localization was successful with either accelerometers or strain sensors, but not both. (9) Damage localization to a reconstructed virtual sensor was possible. (10) Damage detection performance was slightly higher using the stored and reconstructed data than all virtual sensors, but generalization of this result remained questionable and needs further investigation. Different damage types should be studied with a more complex structure. Experimental results are also needed to validate the proposed technique.

## Figures and Tables

**Table 1:** Damage scenarios in a single beam element along a length of 62.5 mm. The measurement numbers are also given.

Damage Level	Plate Thickness mm	Thickness Decrease mm	Measurements
D_0_	5.0	0	1–100
D_1_	4.5	0.5	101–106
D_2_	4.0	1.0	107–112
D_3_	3.5	1.5	113–118
D_4_	3.0	2.0	119–124
D_5_	2.5	2.5	125–130

**Table 2:** Damage detection and localization using raw data. The number in parentheses is the sensor found by the algorithm.

Damage Location	Nearest Sensors	Acceleration Detection	Acceleration Localization	Strain Detection	Strain Localization
Loc 1	1	D5	Fail (5)	D3–D5	OK (1)
Loc 2	15–16		Fail (22)		Fail 23
Loc 3	21–22		Fail (26)	D2–D5	OK (22)
Loc 4	28–29		OK (28)		Fail 16
Loc 5	51–52		OK (51)		Fail 1
Loc 6	22–23		OK (22)		Fail 59

**Table 3:** Damage detection and localization using virtual sensor data. The number in parentheses is the sensor found by the algorithm.

Damage Location	Nearest Sensors	Acceleration Detection	Acceleration Localization	Strain Detection	Strain Localization
Loc 1	1	D1–D5	Fail (3)	D1–D5	OK (1)
Loc 2	15–16		OK (16)	D3–D5	Fail (21)
Loc 3	21–22	D2–D5	Fail (25)	D1–D5	OK (22)
Loc 4	28–29	D3–D5	OK (28)	D4–D5	Fail (38)
Loc 5	51–52	D3–D5	OK (52)	D5	Fail (58)
Loc 6	22–23	D3–D5	Fail (26)	D3–D5	Fail (17)

**Table 4:** Damage detection and localization using stored and reconstructed virtual sensor data. The number in parentheses is the sensor found by the algorithm.

Damage Location	Nearest Sensors	Acceleration Detection	Acceleration Localization	Strain Detection	Strain Localization
Loc 1	1	D1–D5	Fail (3)	D1–D5	OK (1)
Loc 2	15–16	D5	OK (15)	D3–D5	Fail (23)
Loc 3	21–22	D2–D5	Fail (19)	D1–D5	OK (22)
Loc 4	28–29	D2–D5	OK (28)	D2–D5	Fail (21)
Loc 5	51–52	D2–D5	OK (52)	D3–D5	Fail (59)
Loc 6	22–23	D1–D5	Fail (19)	D2–D5	OK (23)

**Table 5:** Damage detection and localization using MD and MMSE algorithms. (Acc = acceleration; raw = raw data; vs = virtual sensor data; rec = stored and reconstructed data). The number in parentheses is the sensor found by the algorithm.

Data	MD Detection	MMSE Detection	MMSE Localization
Acc raw	D5	D5	Fail (4)
Acc VS	D2–D5	D1–D5	Fail (3)
Acc rec	D1–D5	D1–D5	Fail (3)
Strain raw	D5	D5	OK (1)
Strain VS	D2–D5	D1–D5	OK (1)
Strain rec	D1–D5	D1–D5	OK (1)
